# Optimizing Ultrasonic-Assisted Extraction Process of *Paralepista flaccida*: A Comparative Study of Antioxidant, Anticholinesterase, and Antiproliferative Activities via Response Surface Methodology and Artificial Neural Network Modeling

**DOI:** 10.3390/molecules30163317

**Published:** 2025-08-08

**Authors:** Mustafa Sevindik, Ayşenur Gürgen, Aras Fahrettin Korkmaz, Ilgaz Akata

**Affiliations:** 1 Department of Biology, Faculty of Engineering and Natural Sciences, University of Osmaniye Korkut Ata, 80000 Osmaniye, Türkiye; 2 Department of Industrial Engineering, Faculty of Engineering and Natural Sciences, University of Osmaniye Korkut Ata, 80000 Osmaniye, Türkiye; aysenurgurgen@osmaniye.edu.tr; 3 Dietetics Department, Faculty of Health Sciences Nutrition, Şirinevler Campus, İstanbul Kültür University, 34191 Istanbul, Türkiye; a.korkmaz@iku.edu.tr; 4 Department of Biology, Faculty of Science, University of Ankara, 06000 Ankara, Türkiye

**Keywords:** extraction optimization, artificial intelligence, RSM, ANN-GA, antioxidant activity, anticholinesterase, antiproliferative effect

## Abstract

In this study, extraction conditions were optimized to maximize the biological activities of extracts obtained from *Paralepista flaccida*, an edible mushroom species. Extraction processes were carried out using an ultrasonically assisted system, and two different optimization approaches were used as follows: Response Surface Methodology (RSM) and Artificial Neural Network–Genetic Algorithm (ANN-GA). The antioxidant potentials of the optimized extracts were evaluated using DPPH, FRAP, TAS, TOS, and OSI parameters; anticholinesterase activities were measured against AChE and BChE enzymes; and antiproliferative activities were investigated in A549, MCF-7, and DU-145 human cancer cell lines. In addition, phenolic contents were determined by LC-MS/MS analysis. The findings revealed that the extracts obtained by the RSM method exhibited a superior biological profile compared to ANN-GA extracts in terms of antioxidant, anticholinesterase, and antiproliferative activities. The high cytotoxicity observed, particularly in the MCF-7 line, supports the anticancer potential of this extract. These results demonstrate that optimization strategies are crucial for increasing not only extract yield but also biological functionality.

## 1. Introduction

Medicinal mushrooms, despite their widespread nature, are among the most valuable natural resources in healthcare due to their biologically active compounds. Used for centuries in traditional medicine to treat various diseases, these organisms are now attracting attention with their versatile pharmacological effects, supported by modern scientific studies [1,2]. They are known to exhibit various biological activities, including anti-inflammatory, immunomodulatory, antioxidant, and antiproliferative [3]. These pharmacological effects are particularly associated with secondary metabolites such as polysaccharides, phenolic compounds, triterpenes, and sterol derivatives found in the fungal cell walls and metabolic systems [4,5]. Recent research provides scientific data supporting the therapeutic potential of these compounds, making medicinal mushrooms a valuable bioresource for functional foods, pharmaceutical products, and biotechnological applications [6].

In this context, *Paralepista flaccida* (Sowerby) Vizzini (Old name: *Lepista flaccida*), selected as the material of the study, is an edible wild mushroom species that has come to the fore in the scientific literature in recent years due to its nutritional content and biological effects. Studies on its chemical composition, antioxidant capacity, and taxonomic structure, in particular, support the potential for evaluating this species in the field of health. Rich in phenolic compounds, *P. flaccida* has a moderate flavonoid content. It also contains compounds with antioxidant properties such as ascorbic acid, tannins, and carotenoids. In addition to the main components such as glycerol and mannitol, its chemical composition has been reported to include various sugars, amino acids, fatty acids, and organic acids. Its antioxidant potential was evaluated by methods such as DPPH, β-carotene bleaching, and ferric reduction tests, and it was reported to show significant activity in these tests [7,8]. With these features, *P. flaccida* stands out as a species that can be evaluated among natural antioxidant sources.

In this study, extraction conditions were determined using artificial intelligence-based optimization methods to maximize the biological activity of extracts obtained from *P. flaccida* samples. First, key parameters affecting the extraction process, such as temperature, time, and solvent ratio, were optimized using Response Surface Methodology (RSM) and Artificial Neural Network–Genetic Algorithm (ANN-GA) approaches. Extracts obtained under the determined optimum conditions were evaluated for antioxidant, anticholinesterase, and antiproliferative activities, and their phenolic contents were also analyzed in detail. The findings demonstrate the effectiveness of AI-assisted optimization in enhancing the biological effects of *P. flaccida* and support the potential of this species for pharmaceutical applications. Therefore, this study aimed to optimize the extraction conditions of *P. flaccida* using both RSM and ANN-GA, with a particular focus on TAS as a guiding response parameter. Comparative analyses of the resulting extracts were conducted to evaluate and enhance TAS-based extraction efficiency and the related biological activities.

## 2. Results and Discussion

### 2.1. Optimization of Extraction Conditions

In this study, the effects of three key extraction parameters on Total Antioxidant Status (TAS) were investigated in detail. The independent variables examined included extraction temperature (30, 45, and 60 °C), application time (30, 45, and 60 min), and the ethanol/water mixture (defined as the solvent ratio) (0%, 50%, and 100%). The TAS values obtained from extraction processes based on different combinations of these parameters are presented comprehensively in Table 1. The findings were evaluated using both comparative statistical tests and multivariate analysis techniques, thus demonstrating the effect of each variable on TAS in a scientifically meaningful and interpretable manner.

The obtained TAS data clearly demonstrated the effects of the applied extraction conditions on antioxidant capacity. In particular, the extraction process performed at 45 °C, for 45 min and with a 50% ethanol/water ratio yielded the highest TAS value of 3.945 ± 0.022 mmol/L, demonstrating that this parameter combination allows for the efficient extraction of phenolic compounds. At the same temperature, when the extraction time was extended to 60 min and the solvent was changed to consist solely of ethanol, the TAS value decreased to 2.543 ± 0.017 mmol/L. This decrease suggests that both prolonged extraction time and a high ethanol content may have negative effects on the stability of antioxidant compounds.

A more significant decrease in TAS levels was observed with an increase in temperature to 60 °C. The values measured at this temperature ranged from 2.170 ± 0.023 mmol/L (60 min, 0% ethanol) to 3.438 ± 0.031 mmol/L (45 min, 50% ethanol), indicating that high temperatures can cause thermal degradation of phenolic compounds and thus negatively affect extraction efficiency. Furthermore, increasing the extraction time to 60 min resulted in a decrease in TAS values not only at 60 °C but at all temperature levels, with this effect becoming more pronounced at higher temperatures.

In contrast, the TAS data obtained at 30 °C showed a more moderate change (2.148–3.647 mmol/L), indicating that relatively lower temperature conditions can better preserve the structural integrity and stability of antioxidant compounds.

Based on the experimental findings, two different optimization approaches were applied in this study. In the modeling process conducted using RSM, four separate regression models were created as follows: linear, two-factor interaction (2FI), quadratic, and cubic, and these models were compared statistically. Analyses revealed that the quadratic model, with a coefficient of determination (R^2^) of 0.992, provided the strongest fit to the available data set.

This high R^2^ value indicates that the model explained approximately 99.2% of the total variance in the dependent variable, TAS. This finding confirms the model’s high predictive power and that it reflects the relationships between the independent variables and the response variable in a statistically significant manner. Furthermore, it was concluded that the model effectively represented the patterns in the data set and was a reliable tool in terms of both predictive accuracy and statistical consistency in optimizing extraction parameters.

The mathematical expression of the quadratic regression model created to estimate TAS values of extracts obtained from the *P. flaccida* species is given as follows:

$$TAS=3.94-0.082\;X_{1}-0.478{\;X}_{2}+0.024\;X_{3}+0.064\;{X_{1}X}_{2}+0.002\;X_{1}X_{3}\;\;-0.012\;X_{2}X_{3}-0.401\;X_{1}^{2}-0.714{\;X}_{2}^{2}-0.151\;X_{3}^{2}$$

The independent variables evaluated in the quadratic regression model consisted of the following three key extraction parameters: extraction temperature (*X*_1_), treatment time (*X*_2_), and ethanol/water ratio (*X*_3_). The effects of these variables on TAS obtained from *P. flaccida* samples were comprehensively examined, considering both the individual contributions of each factor and the interactions between variables.

**Figure 1:**
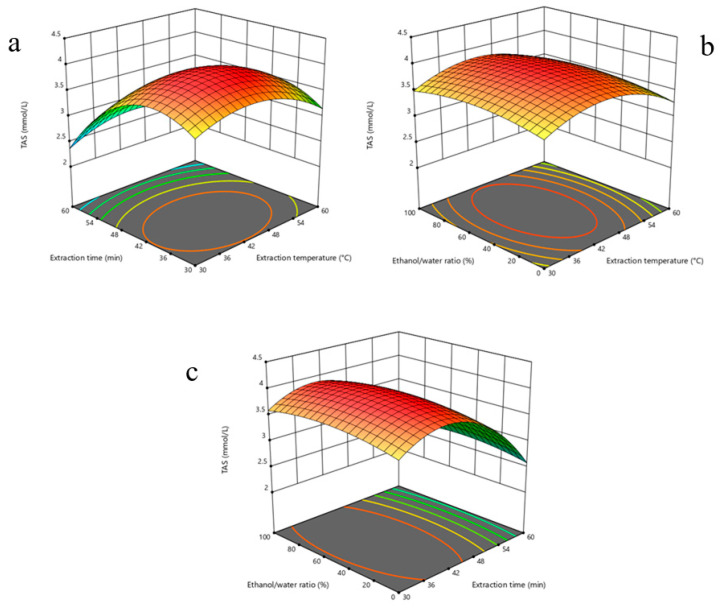
Three-dimensional response surface plots illustrating the interactive effects of extraction parameters on Total Antioxidant Status (TAS) of *P. flaccida* extracts: (**a**) effect of extraction temperature and time at fixed ethanol/water ratio (50%); (**b**) effect of extraction temperature and ethanol/water ratio at fixed extraction time (45 min); (**c**) effect of extraction time and ethanol/water ratio at fixed temperature (45 °C). The plots reveal optimal regions for maximizing TAS values, highlighting significant interactions among variables.

Data obtained from the modeling process were converted into three-dimensional response surface plots (Figure 1) to visually and more clearly reflect the interactions of multiple variables. These plots clearly illustrate the impact of each independent variable on TAS levels at both the individual and interaction levels and serve as an effective guide in determining the most appropriate processing conditions.

In the second phase of the study, the Artificial Neural Network (ANN) method was chosen for the predictive modeling process based on experimental data. During this process, which included a comparative analysis of different neural network architectures, the structure that provided the highest predictive accuracy was identified, and a hybrid model was developed by integrating this architecture with a Genetic Algorithm (GA) to increase optimization capacity. In this context, the 3-6-1 ANN model, consisting of six neurons in a single hidden layer, was identified as the most successful structure according to statistical suitability criteria.

The model’s predictive performance was calculated as follows: Mean Square Error (MSE) 0.003, Mean Absolute Percentage Error (MAPE) 0.377%, and Correlation Coefficient (R) 0.999. These statistical results demonstrate that the model not only provides high-accuracy predictions but also possesses strong generalization capabilities.

**Figure 2:**
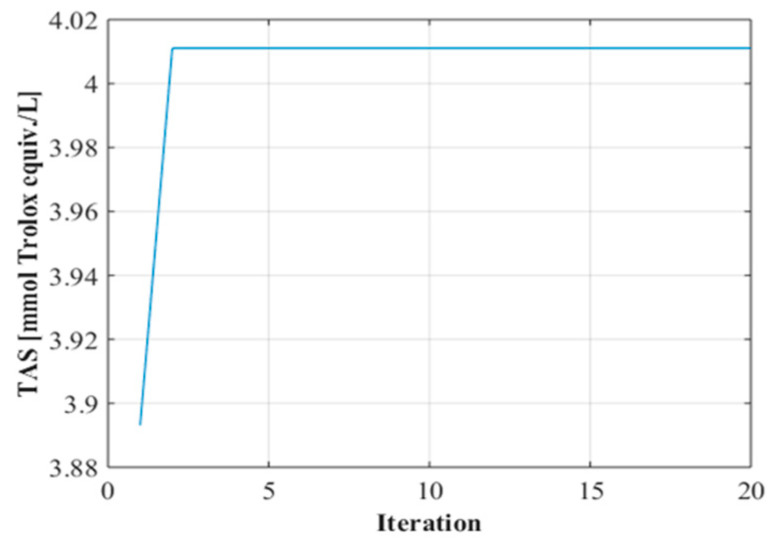
Convergence graph.

In the GA-assisted optimization process, searches were conducted to determine ideal operating conditions based on the most successful prediction results obtained by the ANN model. In this process, population size, one of the critical parameters that directly affects the success of the algorithm, was tested at different levels, and as a result of comparative analyses, the optimum number of individuals was determined to be 5. The findings obtained from the convergence curves (Figure 2) reveal that the algorithm reached a balanced solution after approximately two iterations and the optimization process was successfully completed.

### 2.2. Antioxidant Activity

Mushrooms exhibit significant antioxidant properties thanks to the phenolic compounds, ascorbic acid, flavonoids, and various organic acids they contain [9]. These natural compounds play a role in neutralizing free radicals, contributing to the reduction in oxidative stress and the prevention of cellular damage [10]. In our study, the antioxidant potential of *P. flaccida* mushroom extracts produced at optimal levels was determined. The findings are shown in Table 2.

The antioxidant capacities of optimized extracts obtained from *P. flaccida* were evaluated based on five complementary parameters (DPPH, TAS, FRAP, TOS, and OSI), and the results are presented in Table 2. While both optimization approaches produced biologically active extracts, the extracts obtained with the RSM method exhibited a superior profile compared to ANN-GA in all antioxidant parameters. While the RSM extract demonstrated a higher effect on DPPH radical scavenging capacity, TAS and FRAP results revealed that this extract was richer in components that support the redox system. In contrast, the higher TOS and consequently OSI values in ANN-GA extracts indicate that this method is relatively limited in its ability to suppress oxidative stress balance.

These findings highlight not only the antioxidant activity of the extracts but also the determinant role of extraction parameters in this activity. In the literature, Erbiai et al. [8] reported that *P. flaccida* exhibited significant antioxidant properties using DPPH, β-carotene bleaching, and reducing power tests. However, in this study, the antioxidant potential was evaluated not only in terms of its presence but also in terms of how it differed under optimized conditions, and it was shown that this biological activity could be made more effective, especially with the RSM method. The TAS, TOS, and OSI values of *P. flaccida* have not been reported in the literature. TAS, TOS, and OSI values of different wild mushrooms have been reported. The TAS values of *Otidea onotica*, *Lactarius deliciosus*, *Hericium erinaceus*, and *Cantharellus cibarius* were reported as 8.866, 7.468, 5.426, and 5.511 mmol/L, respectively. TOS values were reported as 14.724, 13.161, 6.621, and 7.289, respectively. OSI values were reported as 0.166, 0.176, 0.122, and 0.132, respectively [11,12,13,14]. In our study, the TAS values of RSM and ANN-GA extracts of *P. flaccida* were determined to be lower than those of *O. onotica*, *L. deliciosus*, *H. erinaceus*, and *C. cibarius*. The TAS value highlights the entirety of the antioxidant compounds produced in natural products [15]. When evaluated in this context, although the total antioxidant capacity of *P. flaccida* extracts is among the species with limited antioxidant capacity, it can be relatively increased, especially with the RSM method. Furthermore, it is understood that antioxidant potential is closely related not only to genetic differences between species but also to the extraction methods used. The TOS values of the RSM and ANN-GA extracts of *P. flaccida* used in our study were determined to be lower than those of *O. onotica* and *L. deliciosus*, but higher than those of *H. erinaceus* and *C. cibarius*. The TOS value highlights the full range of oxidant-active compounds produced in natural products [15]. This suggests that *P. flaccida* extracts have a moderate oxidative load profile and that the oxidant/antioxidant balance can be optimized using the extraction method. The lower TOS values of the extracts obtained using the RSM method, in particular, suggest that this method is more advantageous in reducing compounds that cause oxidative stress.

The OSI values of the RSM and ANN-GA extracts of *P. flaccida* used in our study were determined to be higher than those of *O. onotica*, *L. deliciosus*, *H. erinaceus*, and *C. cibarius*. OSI values indicate the percentage suppression of oxidant compounds produced by the mushroom by antioxidant compounds [15]. This result demonstrates that both optimization methods contribute to maintaining redox balance in biological systems, and that this balance can be improved more effectively, particularly with the RSM method. In conclusion, RSM-based optimization has been shown to maximize not only extract yield but also functional bioactivity, further confirming the strategic importance of method selection in natural product research.

### 2.3. Anticholinesterase Activity

Mushrooms can exhibit anticholinesterase activity due to the various phenolic compounds and secondary metabolites they contain. These properties inhibit acetylcholinesterase (AChE) and butyrylcholinesterase (BChE) enzymes, offering a potential biological defense mechanism against cholinergic dysfunction associated with neurodegenerative diseases [16,17]. In our study, the anticholinesterase activity of the optimized extract of *P. flaccida* was determined. The IC50 values of the obtained results are shown in Table 3.

The anticholinesterase potential of *P. flaccida* extracts was evaluated through their inhibitory effects on acetylcholinesterase (AChE) and butyrylcholinesterase (BChE) enzymes; the data obtained revealed that extracts prepared using both optimization methods were significantly effective against these enzymes. While galantamine, used as a positive control, would be expected to have a stronger inhibitory effect, the multicomponent and natural structure of the mushroom extracts is particularly important for their low toxicity profile and potential for synergistic effects. In this context, the results obtained indicate that naturally occurring inhibitors can be considered for pharmaceutical evaluation, even though they present weaker absolute values compared to artificially synthesized molecules. Furthermore, the fact that the extract optimized with the RSM method exhibited a stronger inhibition profile compared to the ANN-GA method suggests that extraction conditions may be decisive in biological activity and that the optimization strategy may directly affect efficacy. The lack of any previous evidence in the literature regarding the anticholinesterase activity of *P. flaccida* indicates that this study represents a unique contribution to the literature. Furthermore, various previous studies have demonstrated that different fungal species can exhibit significant inhibitory effects on AChE and BChE enzymes [18,19,20]. In this context, the findings obtained in this study indicate that *P. flaccida* could be considered among the natural agents with neuroprotective potential and provide important preliminary data for the discovery of new natural inhibitor candidates, particularly for degenerative diseases related to the cholinergic system. Also, it should be noted that the Ellman assay, although widely used for evaluating acetylcholinesterase inhibition, may yield false positive results under certain conditions due to interference from non-specific reactions, as reported by Drączkowskie et al. [21]. While this method offers simplicity and sensitivity, confirmatory assays such as those involving radiometric or fluorometric techniques were not applied in the current study. This represents a methodological limitation that should be addressed in future investigations.

### 2.4. Antiproliferative Activity

**Figure 3:**
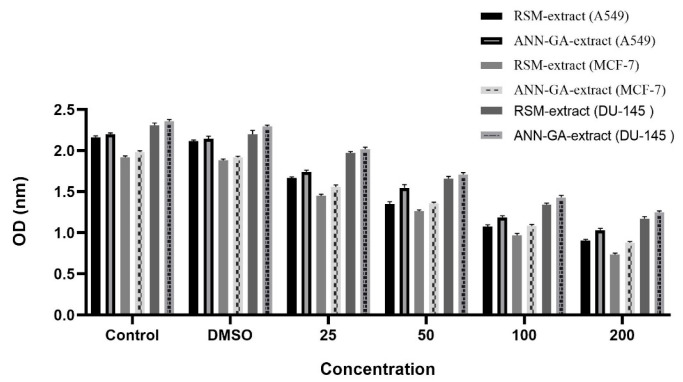
Antiproliferative activity of *Paralepista flaccida* optimized extract.

Mushrooms can exhibit antiproliferative effects on various cancer cell lines through their phenolic compounds, polysaccharides, and terpene derivatives. These natural compounds can suppress cell proliferation and trigger programmed cell death (apoptosis), making them considered promising biological agents for anticancer research [22,23]. In our study, the activities of optimized *P. flaccida* extract against A549, MCF-7, and DU-145 cell lines were determined. The findings are shown in Figure 3.

In Figure 3, the antiproliferative effects of *P. flaccida* extracts at different concentrations were evaluated on three human cancer cell lines, A549 (lung), MCF-7 (breast), and DU-145 (prostate). Cell viability was measured based on OD (optical density) values, with lower OD values being interpreted as indicating a higher antiproliferative effect. According to the findings, the extract optimized by the RSM method at a concentration of 200 µg/mL showed the highest cytotoxic effect on the MCF-7 cell line. This was followed by the effect of ANN-GA extract on MCF-7, the response of RSM extract on A549 line, the effect of ANN-GA extract on A549, the effect of RSM extract on DU-145 line, and finally, the activity of ANN-GA extract on DU-145. This ranking clearly demonstrates that variables such as extraction method and cell type play a decisive role in the biological response. The DMSO group exhibited OD values similar to the control group, confirming the absence of any solvent-induced toxic effects. The antiproliferative effect exhibited by the optimized extracts appears to be directly related to the mushroom extract. In particular, the stronger effects of the extract obtained with the RSM method compared to ANN-GA in MCF-7 and A549 cells further demonstrate the effectiveness of classical statistical optimization strategies in enhancing biological activity. The limited number of studies on *Lepista flaccida* (current name: *P. flaccida*) in the literature contains data on antiproliferative effects limited to the MCF-7 breast cancer cell line, and a moderate cytotoxic effect has been reported in this cell line [24]. In contrast, this study also evaluated both A549 (lung) and DU-145 (prostate) cell lines, and significant antiproliferative effects were observed in these cells. This makes the present study a unique contribution to the literature, not only in terms of extraction optimization but also in terms of the broad range of cells tested. The obtained data demonstrate that *P. flaccida* possesses potential anticancer effects against various cancer types and suggest that optimized extraction conditions can significantly enhance this biological activity. The antiproliferative findings not only demonstrate the impact of extraction parameters on biological activity but also highlight the importance of biological differences between species. In particular, the significant cytotoxic effects of optimized extracts, even at low concentrations, indicate the potential synergistic effects of phenolic compounds, polysaccharides, and other secondary metabolites found in complex mushroom extracts. This suggests that, unlike single-component drugs, multicomponent natural products can elicit multifaceted biological responses in target cells. Furthermore, the comparative application of artificial intelligence-based (ANN-GA) and statistical (RSM) modeling techniques in this study provides a methodological contribution to the literature by demonstrating that extraction efficiency and biological activity can be rationally improved. This study further demonstrates that optimization processes can be used as a strategic tool in natural product research, not only for chemical content but also for maximizing biological functions.

### 2.5. Phenolic Contents

Mushrooms are natural organisms rich in phenolic compounds, and these compounds form the basis of their antioxidant and therapeutic effects. Phenolic acids, flavonoids, and their derivatives, in particular, play a decisive role in the biological activities of mushroom extracts [25]. In our study, the phenolic contents of the optimized *P. flaccida* extract were screened using an LC-MS/MS device. The findings are shown in Table 4.

In this study, the quantitative composition of phenolic compounds in optimized extracts of *P. flaccida* was analyzed in detail, and the effects of both extraction methods (RSM and ANN-GA) on the extract profiles were evaluated (Table 4). The results revealed that redox-active phenolics, particularly gallic acid, 2-hydroxycinnamic acid, protocatechuic acid, and caffeic acid, were present at higher levels in the RSM extract. These compounds are thought to contribute significantly to the antioxidant and potential therapeutic properties of the mushroom. The amount of gallic acid in the extract obtained by the RSM method was measured as 7.27 mg/g, which was significantly higher than in the ANN-GA extract. Similarly, the RSM extract exhibited a richer profile than the ANN-GA in compounds such as 2-hydroxycinnamic acid (5.17 mg/g), protocatechuic acid (2.17 mg/g), and caffeic acid (2.33 mg/g). This difference demonstrates that extraction parameters play a decisive role in phenolic recovery, and that optimized conditions directly impact the biological effect profile.

Comparison of phenolic profiles with previous research also reveals noteworthy findings. In a study conducted by Erbiai et al. [8], the phenolic content of *P. flaccida* was examined using HPLC analysis, and the highest levels of p-hydroxybenzoic acid (138.5 µg/g) were reported. Chlorogenic acid, gallic acid, cinnamic acid, catechin, and protocatechuic acid were subsequently identified, respectively. The phenolic standards used in our study were selected based on their known occurrence in fungal species, including *P. flaccida*, and their documented biological importance (e.g., antioxidant and therapeutic activity). Among the compounds identified in our study, gallic acid, caffeic acid, protocatechuic acid, and p-hydroxybenzoic acid were previously reported in *P. flaccida* [8], validating our screening approach. However, in our study, phenolics such as gallic acid, 2-hydroxycinnamic acid, caffeic acid, and quercetin were detected at much higher concentrations in the same species, demonstrating the effectiveness of the applied optimization strategies (especially RSM) in increasing extraction efficiency. Furthermore, to our knowledge, compounds such as quercetin and 2-hydroxycinnamic acid have not been previously reported in this species. This result suggests that our extraction and detection protocol allows for a broader phytochemical characterization. These results expand the current understanding of the phenolic spectrum of *P. flaccida* and highlight its potential as a rich source of bioactive molecules. The detection of some phenolics, such as quercetin, not detected in the literature, at higher concentrations in our study suggests that environmental and technical factors, such as sample collection time, geographical variation, solvent system used, and extraction methods, are determinants of the phenolic compound profile.

Overall, this study demonstrated that *P. flaccida* not only possesses a rich phenomenon, but also that these bioactive compounds can be maximally recovered through optimized extraction methods. This strengthens the mushroom’s potential as a source of functional foods, natural antioxidants, and pharmacological agents. It can be said that the RSM technique, in particular, stands out for its ability to enhance biological effects in phenolic extraction and offers a strategic advantage in future bioprocess designs.

## 3. Materials and Methods

### 3.1. Extraction Procedure Method

**Figure 4:**
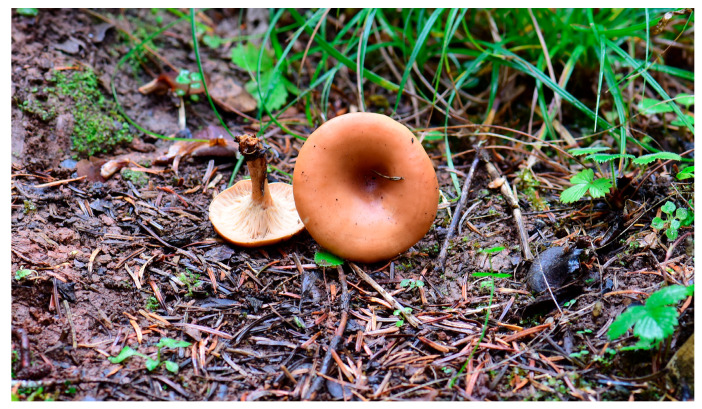
Morphological appearance of *Paralepista flaccida* fruiting bodies collected from a natural habitat.

Mushroom samples used in the study were collected from Antalya (Turkey) (Figure 4). Fungarium samples of the fungus are preserved in the Fungarium of Ankara University. In this study, the effects of three basic parameters were systematically evaluated in order to maximize the extraction efficiency of samples of the *P. flaccida* species. Mushroom samples were collected from Antalya region in 2024, and after they were transported to the laboratory, they were sliced and dried in a fruit dryer (WMF KITCHENminis, Geislingen, Germany) at 45 °C for 48 h. After the drying process, 5.00 g of dried mushroom material for each experimental condition was taken into the extraction process with 100 mL of solvent. Three independent variables were defined in the experimental design as extraction temperature, application time, and ethanol/water ratio; each of these variables was examined at three different levels to form a full factorial matrix including a total of 27 conditions. Temperature levels were 30, 45, and 60 °C; durations were 30, 45, and 60 min; solvent ratios were set at 0%, 50%, and 100% ethanol. All extractions were performed in an ultrasonic bath (WIGGENS UA22MFDN, Wuppertal, Germany) with a frequency of 40 kHz, 100% power, and a capacity of 400 W. The resulting extracts were filtered through Whatman No. 1 filter paper, and the solvents were evaporated under vacuum at 40 °C. Each experimental condition was applied in triplicate (*n* = 3), and the resulting extracts were stored at +4 °C for further analysis.

Data obtained from the experiments were analyzed using Response Surface Methodology (RSM). Interactions between parameter combinations were statistically evaluated, and optimal conditions were determined. Furthermore, Artificial Neural Network (ANN) and Genetic Algorithm (GA) methods were incorporated into the modeling phase, enabling detailed analysis of the simultaneous effects of parameters. In this way, complex relationships between variables have been analyzed more deeply, and a scientific basis has been provided for artificial intelligence-supported decision processes.

### 3.2. Standards and Chemicals

All analytical-grade chemicals and standards used in this study were purchased from Sigma-Aldrich (St. Louis, MO, USA) unless otherwise stated. These included 2,2-diphenyl-1-picrylhydrazyl (DPPH), Trolox, 2,4,6-tripyridyl-s-triazine (TPTZ), 5,5′-dithiobis(2-nitrobenzoic acid) (DTNB), acetylthiocholine iodide (ATCI), butyrylthiocholine iodide (BTCI), galantamine hydrobromide, Folin–Ciocalteu reagent, and phenolic acid standards. All solvents used for extraction and LC-MS/MS analysis (ethanol, methanol, and formic acid) were of analytical grade and suitable for LC-MS/MS analysis.

### 3.3. Response Surface Methodology (RSM)

In this study, Response Surface Methodology (RSM) was used as the basis for determining the optimal levels of key parameters affecting the extraction process. In the experimental design, extraction temperature, application time, and ethanol/water ratio were considered as independent variables, while the Total Antioxidant Status (TAS) of the extracts obtained with different combinations of these variables was evaluated as the dependent variable (response). Modeling and optimization processes were carried out using Design Expert 13 software, and experimental data were analyzed parametrically within the scope of the quadratic polynomial regression model. The mathematical model developed in this direction was established in accordance with the general equation structure presented as follows:

$$Y_{k}=\beta_{k0}+\sum_{i=1}^{n}\beta_{ki}x_{i}+\sum_{i=1}^{n}\beta_{kii}x_{i}^{2}+\sum_{i=1}^{n-1}\sum_{j=i+1}^{n}\beta_{kij}x_{i}x_{j}$$

The dependent variable *Y_k_* in the model represents the TAS values of the extracts obtained under each experimental condition. The terms *X_i_* used as independent variables represent the coded forms of parameters such as extraction temperature, application time, and solvent ratio (ethanol/water). *β*_0_, the constant term of the regression model, reflects the reference level corresponding to the center point in the experimental design. To assess the validity and statistical relevance of the established model, a detailed statistical analysis was conducted, taking into account basic statistical criteria such as coefficient of determination (R^2^), analysis of variance (ANOVA), and *p*-value. During the model optimization process, the resulting mathematical equation was derived to identify critical points, thus achieving the most appropriate TAS values. Furthermore, three-dimensional response surface plots were used to visualize both the individual effects of the independent variables and their interactions. The evaluations made on these plots were integrated with the experimental findings and analyzed in detail within a scientific framework.

### 3.4. Artificial Neural Network–Genetic Algorithm (ANN-GA)

In this study, the Artificial Neural Network (ANN) method was used in the predictive modeling process. Extraction temperature, processing time, and ethanol/water ratio were considered as input variables, while TAS was used as the output (response) variable. To increase the predictive power of the model and test its overall validity, the data set was randomly divided into 80% training, 10% validation, and 10% test subgroups. The learning process was conducted using the Levenberg–Marquardt (LM) algorithm, known for its high processing speed and low error rate. To determine the optimal structure of the neural network architecture, systematic analyses were conducted by creating different topologies with the number of neurons in the hidden layer ranging from 1 to 20. The learning rate and momentum coefficient were kept constant at 0.5 in all configurations; the maximum iteration number for the training process was 500; the early stopping criterion was set at 50 iterations; and the error tolerance was set at 1 × 10^−5^. Each network architecture was run independently 1000 times, and the results were compared against multiple performance indicators. The following two primary error metrics were used to evaluate the model’s numerical performance: Mean Square Error (MSE) and Mean Absolute Percentage Error (MAPE). These metrics were calculated based on the following mathematical formulas:

(1) $$MSE=\;\frac{1}{n}\sum_{i=1}^{n}{\left(e_{i}-p_{i}\right)}^{2}\;$$

(2) $$MAPE=\frac{1}{n}\sum \left|\frac{e_{i}-p_{i}}{e_{i}}\right|*100\;\;$$

In the error analysis formula used in the modeling process, the term e_i_ represents the observation results obtained from experimental data, and pi represents the values predicted by the artificial intelligence-based model. The parameter n here represents the total number of observations analyzed.

The Genetic Algorithm (GA) was chosen as the analysis algorithm during the computational optimization phase, which was conducted to determine the most suitable processing conditions. To evaluate the algorithm’s predictive success, various scenarios were designed under different population sizes, and the impact of this variable on model outputs was demonstrated through comparative analyses. A probability-based “roulette wheel selection” strategy was used during the selection phase of new individuals, and a single-point crossover method was also applied to expand the search space while preserving genetic diversity.

The iterative nature of the GA was visually observed and interpreted through the convergence plots obtained throughout the process. These plots clearly demonstrate the impact of increasing the number of iterations on optimization success. Additionally, each GA scenario is designed to contain at least 30 independent iterations to increase the probability of the algorithm reaching global optimum solutions and strengthen its overall consistency.

### 3.5. Extraction for Bioactivity

In this study, optimal extraction conditions were determined to maximize the biological activity of extracts obtained from mushroom samples, and production was carried out using parameters closest to these conditions. Optimization using RSM yielded ideal conditions of 43.871 °C, a time of 37.657 min, and a 50.443% ethanol/water ratio. In the hybrid optimization approach using ANN-GA, the optimal values were determined as 39.258 °C, a time of 51.110 min, and a 19.277% ethanol/water ratio. Parameters closest to the predicted values by both methods were obtained using an ultrasonic bath (WIGGENS UA22MFDN, Wuppertal, Germany) with a frequency of 40 kHz, 100% power, and a capacity of 400 W. Extracts produced under these conditions were used in the study’s biological activity analyses.

### 3.6. Antioxidant Activity Tests

#### 3.6.1. Total Antioxidant and Oxidant Analysis

The total antioxidant capacity of mushroom extracts obtained under optimized extraction conditions was determined using the TAS kit developed by Rel Assay Diagnostics, and the results were expressed in mmol Trolox equiv./L. The TAS assay is based on the suppression of color formation resulting from the reaction between antioxidant compounds and the ABTS^+^ radical cation, which reflects the total antioxidant capacity of the sample. The TOS kit from the same company was used to determine total oxidant levels; the data obtained from this analysis were evaluated in μmol H_2_O_2_ equiv./L [26,27].

The Oxidative Stress Index (OSI), used to indicate the level of oxidative stress, was calculated by dividing the total oxidant capacity value by the total antioxidant capacity [28].

#### 3.6.2. DPPH Free Radical Scavenging Activity

The DPPH (2,2-diphenyl-1-picrylhydrazyl) radical scavenging test was used to determine the antioxidant capacity of mushroom extracts. For this purpose, stock solutions of each extract were prepared in dimethylsulfoxide (DMSO) at a concentration of 1 mg/mL. 1 mL of these prepared solutions were homogeneously mixed with 4 mL of 0.004% methanol mixture containing 160 µL of 0.267 mM DPPH solution. The resulting mixture was incubated at room temperature in the dark for 30 min. After incubation, the absorbance values of the mixtures were measured at a wavelength of 517 nm, and the obtained data were expressed as mg Trolox equivalents per gram extract (mg TE/g extract) [29].

#### 3.6.3. Ferric Reducing Antioxidant Power Assay

Ferric reducing capacities of mushroom extracts obtained under optimized extraction conditions were determined based on the FRAP (Ferric Reducing Antioxidant Power) method. For this purpose, 100 µL of stock solutions of each extract were prepared and mixed with 2 mL of FRAP reagent. FRAP reagent was obtained by mixing 300 mM acetate buffer (pH 3.6), 20 mM FeCl_3_·6H_2_O dissolved in 40 mM HCl, and 10 mM 2,4,6-tris (2-pyridyl)-s-triazine (TPTZ) solution at a ratio of 10:1:1. The prepared mixture was incubated at 37 °C for 4 min and then absorbance measurement was performed at a wavelength of 593 nm. The data obtained were finalized by calculating in mg Trolox equivalents per gram extract (mg TE/g extract) [29].

### 3.7. Anticholinesterase Activity Test

The anticholinesterase activities of mushroom extracts obtained under optimized extraction conditions were evaluated using the colorimetric method developed by Ellman and colleagues [30]. Galantamine was used as a reference inhibitor for comparison in the analyses. For this purpose, stock solutions were prepared from mushroom extracts at concentrations ranging from 200 to 3.125 μg/mL. In the experimental setup, 130 μL of 0.1 M phosphate buffer adjusted to pH 8.0 was first added to each microplate well. Then, 10 μL of extract solution and 20 μL of acetylcholinesterase (AChE) or butyrylcholinesterase (BChE) enzyme solution were added. The mixture was incubated at 25 °C in the dark for 10 min. Following the incubation period, 20 μL of DTNB solution [5,5′-dithiobis-(2-nitrobenzoic acid)] and 20 μL of substrate (acetylcholine iodide or butyrylcholine iodide) were added to each well to initiate the reaction. Absorbance values were measured at 412 nm; enzyme inhibition rates and half-maximal inhibitory concentration (IC_50_) values (in μg/mL) were calculated and evaluated based on the obtained data.

### 3.8. Antiproliferative Activity Test

The antiproliferative effects of optimized mushroom extracts were investigated on the following three different human cancer cell lines: A549 (lung adenocarcinoma), MCF-7 (breast adenocarcinoma), and DU-145 (prostate carcinoma). All cell lines were commercially purchased from American Type Culture Collection (ATCC) (A549: CCL-185; MCF-7: HTB-22; DU-145: HTB-81). Working solutions of the extracts were prepared at concentrations of 25, 50, 100, and 200 μg/mL. When the cells reached 70–80% density, they were detached from the surface using 3.0mL Trypsin-EDTA solution (Sigma-Aldrich, MO, USA) and transferred to appropriate culture plates. After seeding, a 24 h incubation was applied to allow the cells to attach to the surface. Then, the relevant extract solutions were added to the cell cultures and incubated for another 24 h. After the incubation process was completed, the culture medium was removed and MTT solution at a concentration of 1 mg/mL was added to each well and the incubation was continued at 37 °C until the formation of purple formazan crystals was observed. The formed crystals were dissolved using dimethylsulfoxide (DMSO) (Sigma-Aldrich, MO, USA). DMSO was also used as a negative control group and was applied in equal volumes to all experimental groups. Absorbance values related to cell viability were measured using an Epoch spectrophotometer (BioTek Instruments, Winooski, VT, USA) at a wavelength of 570 nm [31].

### 3.9. Phenolic Analysis

The phenolic composition of mushroom extracts obtained under optimized extraction conditions was determined using a Shimadzu LCMS-8030 (Kyoto, Japan) triple quadrupole system equipped with an electrospray ionization (ESI) source. Analyses were carried out in both positive and negative ionization modes using the Multiple Reaction Monitoring (MRM) technique to ensure high specificity and sensitivity. Chromatographic separation was performed on an Inertsil ODS-4 C18 analytical column (2.1 × 50 mm, 2 µm), maintained at 40 °C. The mobile phase consisted of solvent A (water with 0.1% formic acid) and solvent B (methanol with 0.1% formic acid), delivered at a constant flow rate of 0.400 mL/min. The gradient elution program was as follows: 5% B from 0.00 to 4.00 min; increased to 95% B from 4.01 to 7.00 min; and re-equilibrated to 5% B from 7.01 to 12.00 min. The maximum system pressure was set to 660 bar. An SIL-20ACXR autosampler (Kyoto, Japan) and a CTO-10ASvp column oven were used to ensure precise injections and stable temperature control. Identification of phenolic compounds was based on the comparison of retention times and MRM transitions with those of authentic reference standards. Quantification was performed by constructing external calibration curves for each compound using at least six concentration levels. All calibration curves exhibited excellent linearity, with coefficients of determination (R^2^) greater than 0.995. A total of 24 standard phenolic compounds were screened, including acetohydroxamic acid, kaempferol, fumaric acid, gallic acid, protocatechuic acid, 4-hydroxybenzoic acid, caffeic acid, naringenin, quercetin, luteolin, catechin hydrate, vanillic acid, syringic acid, thymoquinone, resveratrol, myricetin, salicylic acid, phloridzin dihydrate, 2-hydroxycinnamic acid, oleuropein, 2-hydroxy-1,4-naphthoquinone, silymarin, alizarin, and curcumin. All analytical standards were purchased from Sigma-Aldrich (St. Louis, MO, USA), and all solvents used were of analytical grade and compatible with LC-MS/MS analysis. The representative chromatographic profiles of the standard phenolic compounds are presented in Figure S1 (Supplementary Materials), providing supportive evidence for the identification and quantification results.

### 3.10. Statistical Analysis

Statistical analysis of the experimental data obtained in the study was performed using IBM SPSS Statistics 21.0 software (IBM Corp., Armonk, NY, USA). When comparing the observed means between two independent groups, an independent samples *t*-test was used to determine whether the difference between the groups was statistically significant. In analyses comparing three or more groups, one-way analysis of variance (One-Way ANOVA), which takes into account within- and between-group variations, was used. The threshold for statistical significance in ANOVA applications was α = 0.05. If significant differences were detected between groups in the variance analyses, advanced post hoc analyses were performed using Duncan’s Multiple Range Test to determine which group(s) accounted for these differences.

## 4. Conclusions

This study demonstrated that optimization of extraction parameters using both statistical (RSM) and artificial intelligence-based (ANN-GA) approaches significantly influenced the biological activities of *P. flaccida* extracts. Among the tested extracts, those obtained through RSM exhibited superior antioxidant, anticholinesterase, and antiproliferative activities, likely due to more efficient recovery of redox-active phenolics and bioactive secondary metabolites. These findings underline the strategic importance of extraction design, not only for maximizing yield but also for enhancing functional efficacy. The enhanced antiproliferative response observed against MCF-7 and the novel evidence of cytotoxicity against A549 and DU-145 cells suggest that *P. flaccida* may serve as a promising candidate in oncological studies beyond the previously studied breast cancer model. Moreover, the detection of high levels of gallic acid and 2-hydoxycinamic acid in optimized extracts affirms the pharmacological relevance of optimized phenolic profiles. In future studies, fractionation of the active extracts, mechanistic investigations (e.g., apoptosis induction, ROS modulation), and in vivo validation are recommended. Additionally, integrating green extraction technologies with machine learning-assisted process control may further enhance the applicability of *P. flaccida* in functional food, pharmaceutical, and nutraceutical domains.

## Figures and Tables

**Table 1:** TAS values of the extracts obtained in the study.

Experiment Number	Extraction Temperature (°C)	Extraction Time (min)	Ethanol/Water Ratio (%)	TAS (mmol/L)
1	30	30	0	3.270 ± 0.026 ^j^
2	30	45	0	3.470 ± 0.022 ^k^
3	30	60	0	2.148 ± 0.013 ^a^
4	30	30	50	3.460 ± 0.029 ^k^
5	30	45	50	3.647 ± 0.024 ^m^
6	30	60	50	2.337 ± 0.036 ^c^
7	30	30	100	3.273 ± 0.022 ^j^
8	30	45	100	3.551 ± 0.034 ^l^
9	30	60	100	2.271 ± 0.021 ^b^
10	45	30	0	3.528 ± 0.018 ^l^
11	45	45	0	3.746 ± 0.028 ^n^
12	45	60	0	2.670 ± 0.021 ^e^
13	45	30	50	3.739 ± 0.028 ^n^
14	45	45	50	3.945 ± 0.022 ^p^
15	45	60	50	2.721 ± 0.014 ^f^
16	45	30	100	3.565 ± 0.036 ^l^
17	45	45	100	3.834 ± 0.032 ^o^
18	45	60	100	2.543 ± 0.017 ^d^
19	60	30	0	2.938 ± 0.028 ^g^
20	60	45	0	3.267 ± 0.030 ^j^
21	60	60	0	2.170 ± 0.023 ^a^
22	60	30	50	3.170 ± 0.028 ^i^
23	60	45	50	3.438 ± 0.031 ^k^
24	60	60	50	2.338 ± 0.023 ^c^
25	60	30	100	3.105 ± 0.019 ^h^
26	60	45	100	3.272 ± 0.022 ^j^
27	60	60	100	2.336 ± 0.018 ^b^

Note: Means having the different superscript letter(s) in the same column are significantly different (*p* < 0.05) according to Duncan’s multiple range test.

**Table 2:** Antioxidant parameters of *Paralepista flaccida*.

Parameters	RSM Extract Values	ANN-GA Extract Values
DPPH (mg TE/g extract)	137.35 ± 2.08 ^a^	121.34 ± 1.93 ^a^
TAS (mmol/L)	4.054 ± 0.022 ^a^	3.976 ± 0.014 ^b^
FRAP (mg TE/g extract)	99.35 ± 2.32 ^a^	89.50 ± 1.33 ^a^
TOS (µmol/L)	10.352 ± 0.067 ^a^	11.069 ± 0.128 ^b^
OSI (TOS/(TASx10))	0.255 ± 0.001 ^a^	0.278 ± 0.003 ^b^

Note: Values are presented as the mean ± standard deviation (*n* = 3). Different superscript letters (a, b) in the same row indicate statistically significant differences between groups (*p* < 0.05) according to independent samples *t*-test.

**Table 3:** Anticholinesterase activity of *Paralepista flaccida*.

Sample	AChE μg/mL	BChE μg/mL
RSM extract	65.31 ± 1.50 ^b^	107.82 ± 1.29 ^b^
ANN-GA extract	73.39 ± 1.59 ^c^	120.34 ± 1.55 ^c^
Galantamine	7.61 ± 0.20 ^a^	16.47 ± 0.19 ^a^

Note: Values are expressed as the mean ± standard deviation (*n* = 3). Different superscript letters (a, b, c) in the same column indicate statistically significant differences between groups (*p* < 0.05) according to one-way ANOVA.

**Table 4:** Phenolic contents of *Paralepista flaccida*.

Phenolic Compounds	RSM Extract (mg/g)	ANN-GA Extract (mg/g)
Catechinhyrate	1.36 ± 8.75 ^a^	1.26 ± 6.79 ^b^
Vanillic acid	0.38 ± 3.96 ^a^	0.41 ± 2.72 ^b^
Syringic acid	0.57 ± 8.00 ^a^	051 ± 2.71 ^b^
Gallic acid	7.27 ± 8.92 ^a^	6.94 ± 5.27 ^b^
Protocatechuic acid	2.17 ± 1.72 ^a^	1.76 ± 2.20 ^b^
4-hydroxybenzoic acid	3.87 ± 4.22 ^b^	4.01 ± 2.43 ^a^
Caffeic acid	2.33 ± 3.73 ^a^	2.17 ± 1.54 ^b^
2-hydoxycinamic acid	5.17 ± 2.79 ^a^	3.26 ± 1.59 ^b^
Quercetin	2.04 ± 1.27 ^a^	2.00 ± 1.92 ^b^

Note: Values are expressed as the mean ± standard deviation (*n* = 3). Different superscript letters (a, b) in the same row indicate statistically significant differences between RSM and ANN-GA groups (*p* < 0.05) based on independent samples *t*-test.

## Data Availability

If requested, data related to the study may be requested from the corresponding author upon reasonable request.

## Supplementary Materials

The following supporting information can be downloaded at: https://www.mdpi.com/article/10.3390/molecules30163317/s1, Figure S1: The chromatogram of the standards is included in the appendix.

## Abbreviations

The following abbreviations are used in this manuscript:

DMSO	Dimethyl sulfoxide
DTNB	5.5dithiobis-(2-nitrobenzoic acid)
MTT	(3-[4,5-dimethylthiazol-2-yl]-2,5-diphenyl-tetrazolium bromide)
OSI	Oxidative stress index
TAS	Total antioxidant status
TOS	Total oxidant status
