# Supplementary material for: Optimizing Ultrasonic-Assisted Extraction Process of Paralepista flaccida: A Comparative Study of Antioxidant, Anticholinesterase, and Antiproliferative Activities via Response Surface Methodology and Artificial Neural Network Modeling
Source: Molecules. 2025 Aug 8;30(16):3317. doi: 10.3390/molecules30163317 (PMC12388841; doi:10.3390/molecules30163317)
Supplement: Supplementary file 1 [file molecules-3815812-supplementary.pdf › molecules-3815812-supplementary.pdf]

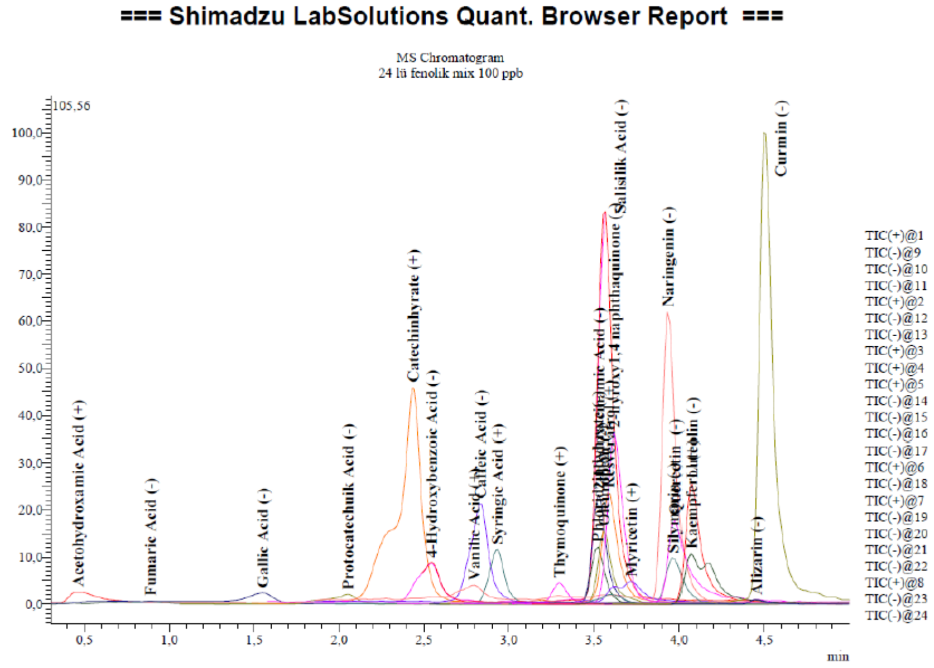

Figure S1. Representative LC-MS/MS Chromatogram of Phenolic Standards Used in This Study

This chromatogram illustrates the retention times and peak distribution of 24 phenolic standard compounds used for the quantification of mushroom extract components. The figure supports the LC-MS/MS-based identification and quantification presented in Table 4 of the manuscript.
